# The PADRE merits of developing and implementing context-specific national action plans for health security in Central Africa: Case study of Cameroon

**DOI:** 10.3389/fpubh.2022.907163

**Published:** 2022-11-24

**Authors:** Frankline Sevidzem Wirsiy, Denis Ebot Ako-Arrey, Claude Ngwayu Nkfusai, Nancy Tahmo, Nicholas Tendongfor, Eugene Vernyuy Yeika, Ndim Eugene Bangwen, Quinta Ngum Ngwa, Luchuo Engelbert Bain

**Affiliations:** ^1^Department of Epidemiology, Emerging Threats Epidemiology Group (ETEG), College of Public Health, University of Nebraska Medical Center, Omaha, NE, United States; ^2^Global Action for Public Health Services [GAPS], Buea, Cameroon; ^3^Catholic Relief Services (CRS), St. Baltimore, MD, United States; ^4^Malaria Consortium, Buea, Cameroon; ^5^Department of Public Health, School of Nursing and Public Health, University of Kwa-Zulu Natal, Durban, South Africa; ^6^Department of Public Health and Hygiene, Faculty of Health Sciences, University of Buea, Buea, Cameroon; ^7^Department of Public Health, Institute of Tropical Medicine, Antwerp, Belgium; ^8^Global South Health Research and Services, GSHS, Amsterdam, Netherlands; ^9^College of Social Science, Lincoln International Institute for Rural Health (LIIRH), University of Lincoln, Lincoln, United Kingdom; ^10^Department of Psychology, Faculty of Humanities, University of Johannesburg, Johannesburg, South Africa

**Keywords:** context-specific, NAPHS, PADRE, merits, Cameroon

## Background

Health is the greatest asset of a state and nothing is more fundamental to a state's security than the health of its people. If individuals and groups of people are unhealthy, they cannot work, and this means that they cannot contribute to the country's economy. If they cannot contribute to the economy of the nation, the government cannot provide essential services, and this leaves the country vulnerable to diverse health security threats, with some caused by natural circumstances and others socio-economical. The initial step in bettering the global health security space is evaluating present capacity and identifying gaps in individual countries. One of the most authoritative measures of preparedness for pandemic response is the Global Health Security Index (1), which failed in predicting the appropriate response to the COVID-19 pandemic. New Zealand, for instance, rated 35th and significantly outplayed the USA (rated the most prepared country) when it came to COVID-19 infections and deaths. Understanding the dimensions of health security, informed by context with robust empirical research, is relevant. The PADRE merits reflect the benefits of implementing context-specific NAPHS in ECCAS, thus strengthening global health security, while the Integrated Disease Surveillance and Response (IDSR) framework improves the identification and response to the main causes of sickness, mortality, and disability in African countries by increasing the use of surveillance and laboratory data. Nonetheless, the IDSR framework fits into the PADRE merits in terms of enhancing the detection of threats early. Implementing NAPHS in Cameroon, and the ECCAS in general, with evidence gained from their IDSR framework only goes to strengthen national laboratory systems, surveillance, and reporting, which are critical areas for a country to be able to respond to threats early as stipulated by the 2005 International Health Regulations [IHR] (2).

The JEE process and its tools, refined by WHO as part of IHR, enhanced a monitoring and evaluation framework to actuate the abilities of nations to fight public health threats (2). It has been established that developing the health security capacity through context-specific plans that are essential to handle communicable disease epidemics/outbreaks is an issue for many Sub-Saharan African countries (3). We insist on the adage context-specific because no country is the same, especially the different zones/provinces that make a country. After a JEE has been executed, countries are encouraged to develop NAPHS to mobilize resources and address gaps (4). Health emergencies, for example, the ongoing monkeypox and cholera outbreak in Cameroon including the COVID-19 pandemic, open up weaknesses in the countries' emergency management. To adequately manage these public health emergencies, countries require robust preparedness and strengthened capacity to efficiently respond to these health threats as reflected in their NAPHS. Preparedness, prevention, and early detection, which are the basis of global health security, become momentous if the globe is to avoid another pandemic of this degree taking place. It is of utmost importance for Central African countries to perform their JEEs and develop, implement, and launch their NAPHS. These NAPHS should be grounded on the One Health paradigm (3). Multicontextual One Health paradigms are decisive to accomplishing better dimensions for health security, given the tremendous monetary risks of disease epidemics and their extra socio-economic severances. The multi-country monkeypox outbreak and COVID-19 pandemic validate that we are all inevitably linked, such that a communicable disease threat anywhere can frankly be a communicable disease threat all around the globe. A NAPHS has been shown to pull country priorities for health security, bring sectors in sync, identify collaborators, and apportion opportunities for its capacity advancement.

## Cameroon and health security

Health security encompasses the activities and responses of nations to minimize the danger and impact of public health threats. In Cameroon, health security is not covered under the current social security protection system. Global Health Security Agenda (GHSA) stipulates that risk plans should be prepared as a key response strategy to the many risky events relating to health emergencies (Health sector strategy plan) (5). In this opinion, which is intended to serve as an advocacy tool for global health security, we examine the merits of implementing context-specific NAPHS in the Central African region connoted as ECCAS with the case study of Cameroon.

Taking into account the JEE results of the six WHO Regions, most of the indicators had an average score of <4, implying that nations were unable to exhibit their amplitude for IHR application (6). African countries, including the ECCAS region, were those with the bulk of the below achievements. Furthermore in Cameroon, health security in the past five decades has been highlighted by many events akin to emerging and re-emerging infectious diseases (7), such as (i) Cameroon experiencing over 50 recurrent cholera epidemics between 1971 and 2022 with high mortality rates; (ii) outbreaks of measles with the occurrence of 38–40 outbreaks per year including those imported in Northern Cameroon in 2016; and (iii) wild poliovirus outbreaks in many parts of the Cameroon territory (8). Moreover, outbreaks of avian influenza in 2006, 2016–2017, 2021, and 2022, and monkeypox in 2016 (7), as well as the recent monkeypox 2022 outbreak, have occurred in Cameroon. It is also worthy to note that, of the six neighboring countries of Cameroon, Gabon, Nigeria, and the Republic of the Congo have recorded Ebola outbreaks. In Cameroon, other major risky events according to the Cameroon National Contingency Plan (9) were the intoxication of animals and humans by gas emissions in 1986 from Lake Nyos and Lake Monoun in 1984; the Mount Cameroon (South West Region) volcanic eruptions in 1982, 1999, and 2000. Also, in 1998, in the Nsam area in Yaounde, a dangerous fire outbreak occurred causing huge human injury, especially that of a truck carrying fuel (9), and the terrific train accident in a locality called Eseka, Center Region of Cameroon, in 2016 (10). We cannot count the numerous fire outbreaks that occurred in Cameroon, especially in market areas, and the fire explosion that hit the production unit of Cameroon's National Refining Company (SONARA) in Limbe, 2019.

For the last 5 years, Cameroon has been dealing with civil unrest in the South West and North West Regions, pushing the massive relocation of persons and increased potential for disease spread, coupled with a long battle against extremist groups like Boko Haram in the Far North and the upsurge of refugees from the Republic of Central Africa in the East Region (11). Based on the above facts, Cameroon must be adequately prepared as far as health security risks are concerned. For this to happen effectively and efficiently, the context-specific NAPHS of Cameroon needs to be concretized and implemented to assist its health systems to be strengthened.

## The PADRE merits of a NAPHS in Cameroon

We used the acronym PADRE to summarize the important aspects of a unified health security plan. PADRE merits shed light on an important aspect, which is that of enhancing the development of a harmonious health security training education program in Cameroon that reflects the realities of its health systems. A crucial element in preventing outbreaks from spreading into epidemics is building frontline capacities through an integrated public health security training program. The United States government through its United States Agency for International Development (USAID) and the Center for Disease Control and Prevention (CDC), as well as other multi-lateral partners, continue to support global health security agendas worldwide (3). In this light, JEE was carried out in Cameroon in 2017 and is in the process of developing a NAPHS. JEE results of Cameroon were established, and it was revealed that its ability to avert the occurrence of health emergencies is either narrow or missing. Also, states in the ECCAS region scored 1 or 2 in the “Prevent” category for the majority of indexes with Cameroon showing limited abilities for a systematic multisectoral analysis of IHR related to biosecurity training, antimicrobial resistance, and biosafety training including its legislations (7). This situation is similar to that of other ECCAS countries where critical gaps were also identified. We further argue that established PADRE merits of NAPHS development and implementation will inform key stakeholders about essential actions that are key to realizing a wider improvement of ECCAS's ability to fight and conquer health risky events in respective countries while minimizing international spread and impact. We present five key merits of implementing NAPHS in ECCAS with a case study of Cameroon which takes into consideration our field experiences and expertise in the operation of GHS projects. The PADRE merits are summarized as follows:

### Preventing avertible outbreaks and/or epidemics

Cameroon, as well as other ECCAS countries, would be able to prevent communicable disease risks from a whole-of-government approach through its context-specific NAPHS. In the same light, NAPHS enhances a nation's supplementary ability for the prevention of infectious disease threats through in-country research mechanisms to develop topical prevention measures, especially diagnostics for identified disease threats and vaccines. To prevent epidemics, countries need their NAPHS to reflect their health systems strongholds with the ability to provide routine services, health infrastructure, and strong veterinary services that can address emerging, re-emerging, and endemic health hazards. Imperative areas of work that NAPHS can focus on within this Prevention category include National Legislation; Antimicrobial Resistance; Financing and Policy IHR Coordination, Advocacy, and Communication; Food Safety; Zoonotic Disease; Biosecurity/Biosafety; and Immunization (12).

### Ameliorating emergency response and preparedness abilities

Ameliorating emergency preparedness and response abilities to bolster healthcare worker systems, surveillance, laboratory, supply chain, and risk communication. Developing and implementing context-specific NAPHS within the ECCAS region and Cameroon, in particular, enables their governments to determine their health security priorities while encouraging partners and donors to fill in gaps identified by the individual country, that is, JEEs. With support from the CDC, WHO, and other partners, nations can yield high-impact and technically sound NAPHS activities, if only they can design the activities to address gaps in health security (3). Collectively, these tools help the government strengthen its health security capacity. Equally, implementing NAPHS enhances profits of domestic support from the apical political levels for health security tasks, and in turn, directs partners to areas where more support is desired.

### Detecting threats early

Developing and implementing context-specific NAPHS within the ECCAS region and Cameroon, in particular, enhances prompt and systematic detection, as well as the broadcasting of communicable disease outbreaks. Public health plans are vital to controlling the sequence of disease outbreaks and hence evading extensive epidemics. In Cameroon, as well as other ECCAS countries, context-specific NAPHS partly based on established JEE results should serve as an example of operating transparency in domestic health security planning for detecting threats early, thus encouraging other Sub-Saharan countries to do the same (2). These context-specific NAPHS activities frame national abilities to uncover, establish, report, and examine health threats in both their animal and human sub-populations. Pertinent areas of work in detecting threats early include Workforce Development, National Laboratory Systems, Surveillance, and Reporting.

### Responding effectively and rapidly

The NAPHS would lay the foundation for ECCAS countries to have multi-partnered emergency strategies in place to counter emerging and re-emerging infectious disease events effectively. Responding effectively and rapidly to infectious diseases and risks of international importance would entail tailored coordination mechanisms among whole-of-government partners (holistic perspective), emergency operations centers, user-context risk communications that are sustainable, concessions between multisectoral partners on human/animal health, law enforcement authorities and security, logical frameworks for personnel deployment, and management of non-medical/medical countermeasures (3). In addition, responding effectively and rapidly is important to manage communicable disease threats at borders and critical to a country's capacity to control these threats from spreading internationally.

### Enhancing the development of unified health security training educational program

A context-specific NAPHS would guide and inform the suitable development and implementation of an integrated public health security training curriculum for ECCAS countries and Cameroon in particular, which in turn raises the magnitude of vigilance for outbreaks in health districts and its facilities and communities. Developing frontline capacities through an integrated public health security training curriculum is a key component for keeping outbreaks from becoming widespread epidemics. CDC remains a key player in helping selected countries (intensive support countries) train and sustain competent and skilled community health workers and/or health professionals (including animal health) using diverse mechanisms in pre- and in-service medical education programs (13). Implementing curricula to promote a multisectoral approach to creating awareness of communicable diseases and expanding in-service public health security training curriculums that address vector as well as zoonotic diseases is critical.

## Conclusion

The PADRE helps identify the benefits of a NAPHS and the steps that countries still need to take to create context-specific NAPHS. Analyzing requirements for donor funding, government, and partners, including other available resources, and performing periodic updates and/or reviews of NAPHS is strategic and important for ECCAS countries. The American CDC is one of those bodies that remain heavily engaged and even with ongoing plans to support these global health security processes of JEE and eventually NAPHS. It happens that many countries have yet to begin JEE process to guide their NAPHS; nevertheless, the fact that even if this assessment is done, it is simply just a part of the bigger picture in terms of steps to strengthening GHS. Planning and implementation of post-JEE NAPHS are vital to the systematic process of building the force toward prevention, detection, and response to communicable disease risks. From a global perspective, the 2017 JEE of Cameroon showed that it had limited preparedness and response capacities, and as such, results should be accumbent in a context-specific NAPHS that is costed and implemented. A powerful socio-political commitment *via* the establishment of multisectoral partners is and remains vital to reinforce IHR's core abilities in Cameroon and ECCAS in general. Building abilities for health security in ECCAS requires different actors to achieve the greatest efficiency through coordinated efforts, thus emphasizing the importance of good leadership. It would be more feasible to guide and implement the NAPHS in Cameroon through the government and dedicated leaders who understand the context, possess adequate technical knowledge, and influence amidst strong political wills.

## Author contributions

FS conceived the study and designed and drafted the original manuscript. FS, DE, CN, NTe, NTa, EV, NE, QN, and LB corrected and improved the original manuscript. All authors read and approved the final manuscript.

## Conflict of interest

Author DE was employed by Catholic Relief Services (CRS). Author CN was employed by Malaria Consortium. The remaining authors declare that the research was conducted in the absence of any commercial or financial relationships that could be construed as a potential conflict of interest.

## Publisher's note

All claims expressed in this article are solely those of the authors and do not necessarily represent those of their affiliated organizations, or those of the publisher, the editors and the reviewers. Any product that may be evaluated in this article, or claim that may be made by its manufacturer, is not guaranteed or endorsed by the publisher.

## Author disclaimer

The authors alone are responsible for the views expressed in this article, which do not necessarily represent the views, decisions, or policies of the institution with which they are affiliated.
